# Cariprazine treatment in patients with borderline personality disorder and psychotic symptoms

**DOI:** 10.1192/j.eurpsy.2025.1926

**Published:** 2025-08-26

**Authors:** P. Perez-Melendez Perez, P. D. S. Calderon, H. Vizcaino Herrezuelo, C. Delgado Marmisa

**Affiliations:** 1 Psychiatry Department, Hospital Universitario Puerta de Hierro, Madrid, Spain

## Abstract

**Introduction:**

Psychotic symptoms are frequently observed in individuals with borderline personality disorder. Numerous case reports in the scientific literature support this finding. This symptoms can significantly impair the patients daily functioning and quality of life. Antipsychotic medications have demonstrated efficacy in managing both psychotic symptoms and emotional dysregulation in this population.

**Objectives:**

This case report aims to explore the use of cariprazine in managing psychotic symptoms in patients with borderline personality disorder.

**Methods:**

This case report describes a 28-year-old woman who sought mental health treatment for symptoms including emotional instability, lability, irritability, anxiety, and impulsivity. Her history includes childhood sexual abuse, which she identified as a significant source of distress. She also reported chronic auditory pseudo-hallucinations with derogatory content and visual pseudo-hallucinations of “small spiders.” She reported these experiences as distressing and disruptive to her daily life. The patient recognized the unreal nature of these hallucinations.

**Results:**

After initiating treatment with cariprazine 2.5mg, the patient demonstrated significant clinical improvement, including a reduction in the severity and impact of psychotic symptoms and a decreased risk of self-harm. Due to this positive response, cariprazine was continued, and the patient experienced increased emotional stability without requiring further acute care.

**Conclusions:**

Studies have suggested the efficacy of second-generation antipsychotics in treating patients with borderline personality disorder. Beyond the remission of psychotic symptoms, these medications can lead to improvements in both affective and behavioral functioning.

**Disclosure of Interest:**

None Declared

